# Randomized clinical trial on the preservation of the medial pectoral nerve following mastectomy due to breast cancer: impact on upper limb rehabilitation

**DOI:** 10.1590/S1516-31802009000300002

**Published:** 2009-11-04

**Authors:** Andrea de Vasconcelos Gonçalves, Luiz Carlos Teixeira, Renato Torresan, César Alvarenga, César Cabello

**Affiliations:** 1 PT, MSc. Physiotherapist, Department of Obstetrics and Gynecology, School of Medical Sciences, Universidade Estadual de Campinas (Unicamp), Campinas, São Paulo, Brazil.; 2 MD, PhD. Associate professor, Department of Obstetrics and Gynecology, School of Medical Sciences, Universidade Estadual de Campinas (Unicamp), Campinas, São Paulo, Brazil.; 3 MD, PhD. Physician, School of Medical Sciences, Universidade Estadual de Campinas (Unicamp), Campinas, São Paulo, Brazil.

**Keywords:** Breast cancer, Mastectomy, Pectoral nerves, Rehabilitation, Physical therapy (specialty), Neoplasias da mama, Mastectomia, Nervos torácicos, Reabilitação, Fisioterapia (especialidade)

## Abstract

**CONTEXT AND OBJECTIVE:**

Systematic modifications to the surgical technique of mastectomy have been proposed with the objective of minimizing injuries to the pectoral nerves and their effects. The aim of this study was to compare muscle strength and mass of the pectoralis major muscle (PMM) and abduction and flexion of the homolateral upper limb following mastectomy among women with breast cancer undergoing either preservation or sectioning of the medial pectoral nerve (MPN).

**DESIGN AND SETTING:**

Randomized, double-blind, clinical trial on 30 women with breast cancer who underwent mastectomy between July 2002 and May 2003 in Campinas, Brazil.

**METHODS:**

The women were allocated to a group, in which the MPN was preserved, or to another group in which it was sectioned. Fisher’s exact and Wilcoxon tests were used to analyze the data, along with Friedman and ANOVA analysis of variance.

**RESULTS:**

In the MPN preserved group, 81% of the women did not lose any PMM strength, compared with 31% in the sectioned MPN group (confidence interval, CI = 1.21; relative risk, RR = 2.14; P < 0.03). There were no differences between the groups regarding muscle mass (CI = 0.32; RR = 0.89; P = 0.8), shoulder abduction (CI = 1.36; RR = 0.89; P = 0.28) and shoulder flexion (CI = 1.36; RR = 1.93; P = 0.8).

**CONCLUSIONS:**

Preservation of the MPN was significantly associated with maintenance of PMM strength, compared with nerve sectioning. No differences in muscle mass or in abduction and flexion of the homolateral shoulder were found between the groups.

**CLINICAL TRIAL REGISTRATION NUMBER:**

ANZCTR - 00082622

## RESUMO

**CONTEXTO E OBJETIVO:**

Modificações sistemáticas técnica cirúrgica das mastectomias têm sido propostas com o objetivo de minimizar lesões dos nervos peitorais e seus efeitos. O objetivo deste artigo foi comparar força e trofismo do músculo peitoral maior (MPM) e amplitude de movimento do membro superior, homolaterais à mastectomia em mulheres com carcinoma de mama submetidas à preservação ou não do nervo peitoral medial (NPM).

**TIPO DE ESTUDO E LOCAL:**

Ensaio clínico aleatório, duplo-cego, com 30 mulheres com carcinoma de mama submetidas a mastectomias entre julho de 2002 e maio de 2003, em Campinas, Brasil.

**MÉTODOS:**

As mulheres foram alocadas em um grupo em que o NPM foi preservado e em outro no qual o NPM foi seccionado. Para análise de dados, foram utilizados os testes exato de Fisher e Wilcoxon, além das análises de variância de Friedman e Anova (análise de variância).

**RESULTADOS:**

No grupo com NPM preservado, 81% das mulheres não sofreram perda de força do MPM comparado a 31% no outro grupo (intervalo de confiança, IC = 1.21 e risco relativo, RR = 2.14, P < 0,03). Em relação a trofismo muscular (IC = 0.32 e RR = 0.89, P = 0.8), a abdução (IC = 1.36, RR = 0.89, P = 0.28) e flexão (IC = 1.36, RR = 1.93 e P = 0.8) do ombro homolateral, não houve diferenças entre os grupos.

**CONCLUSÃO:**

A preservação do MPM foi significativamente associada a manutenção da força do MPM comparada com a secção do nervo. Não foram encontradas diferenças no trofismo muscular ou na amplitude de movimento do ombro entre os grupos.

## INTRODUCTION

In breast cancer surgery, axillary lymphadenectomy and manipulation of the pectoral muscles carries a risk of tissue lesions and general complications in up to 70% of cases, thereby negatively affecting the quality of life of patients.^1^^,^^2^^,^^3^^,^^4^ The complications resulting from axillary lymphadenectomy include chronic pain, limitations to shoulder flexion range of motion, winged scapula, atrophy of the thoracic muscles and shoulder, and paresthesia of the upper limb due to nerve lesions.^2^^,^^4^^,^^5^ In most cases, the pectoral nerves are sectioned during Patey’s surgery.

There are two ways of classifying the pectoral nerves: according to their origin in the brachial plexus or according to the surgeon’s perspective during the procedure of axillary lymphadenectomy.^6^^,^^7^^,^^8^^,^^9^^,^^10^ For the present study, the nomenclature based on their origin in the brachial plexus was used. The lateral pectoral nerve is thus described because it originates in the lateral fascicle of the brachial plexus, while the medial pectoral nerve is so named because it originates in the medial fascicle of the brachial plexus.^9^^,^^10^^,^^11^^,^^12^^,^^13^ The peripheral branches of these nerves are routinely damaged, together with the pectoral muscles, during radical mastectomy. Injury to these nerves during axillary dissection may theoretically result in denervation of the pectoralis major even if this muscle has been preserved during the mastectomy. This denervation might provoke atrophy of the pectoralis major muscle and infraclavicular depression of the thoracic wall, which hampers normal shoulder function and hinders the implantation of a silicone prosthesis or myocutaneous patches during breast reconstruction.^1^^,^^6^^,^^7^^,^^13^^,^^14^^,^^15^

With the objective of minimizing or eliminating these effects, systematic modifications to this surgical technique have been proposed, including preservation of the pectoral nerves to avoid atrophy of the pectoralis major muscle. Some investigators have proposed performing axillary dissection by separating the pectoral muscles.^15^ Other authors have defended the technique of axillary lymphadenectomy using the transpectoral anterior approach from the chest wall.^3^ However, there are very few studies in which pectoral nerve lesions have been correlated with morphofunctional changes in upper limbs. Thus, there is little evidence of advantages in preserving the pectoral nerves. Merson et al.^14^ studied the preservation of the medial pectoral nerve by preserving the pectoralis minor muscle in a modified mastectomy procedure. They reported an atrophy rate of up to 6% in the pectoralis major muscle, compared with a rate of 54% in this muscle in women who underwent removal of the pectoralis minor muscle and the medial pectoral nerve.^14^

## OBJECTIVE

Therefore, based on these concepts, the objective of this study was to evaluate the effect of preservation of the medial pectoral nerve during modified mastectomy procedures, in comparison with its resection, in relation to the strength and mass of the pectoralis major muscle and the range of movement of the homolateral upper limb, among women undergoing a standardized physiotherapeutic rehabilitation program.

## PATIENTS AND METHODS

A randomized, controlled, double-blind clinical trial was carried out involving 30 women with a histological diagnosis of breast cancer and an indication for modified mastectomy. These patients were seen between July 2002 and May 2003, at the Department of Obstetrics and Gynecology, School of Medical Sciences, Universidade Estadual de Campinas (Unicamp), Campinas, Brazil. The protocol was approved by the Institutional Review Board and all of the women signed an informed consent statement before entering the study.

All of the women underwent modified mastectomy. The allocation of the patients was done using sealed envelopes prepared by the computing section of the hospital. In 16 of these women, the medial pectoral nerve was preserved during the mastectomy, while in the remaining 14, this nerve was sectioned during surgery. The routine mastectomy procedure at this service is to section the medial pectoral nerve. To avoid contamination of the groups during the rehabilitation, the incision and consequently the scar was the same in both groups. The physiotherapist was blinded to the kind of surgery that had been performed and the patients were also not informed about the technique.

All of the patients were included in the rehabilitation program at the Physiotherapy Department, which comprised six weeks of follow-up with thrice-weekly sessions and reevaluations 15 and 43 days after surgery. The physiotherapeutic technique used was kinesiotherapy, which consisted of 19 exercises: flexion, extension, abduction, adduction and internal rotation or external rotation of the upper limbs, separately or in combination. The physiotherapeutic procedures also involved techniques and tests to evaluate homolateral upper limb function. Goniometry was used to measure the range of movement of the upper limb; palpation of the pectoralis major muscle compared with sternal insertion was used to evaluate its trophism; and an overload test on the pectoralis major muscle was used to evaluate its strength.

The exclusion criteria were findings of limitations of movement in the homolateral upper limb at the preoperative evaluation; inability to understand the proposed exercises; and occurrences of accidental damage to the medial pectoral nerve during the surgical procedure in women who had been allocated to the preservation group. Women who failed to attend three consecutive physiotherapy sessions were discontinued from the study.

The sample size was estimated as 15 patients in each branch of the study. This was based on the study by Merson et al.,^14^ which found that 54% of the women presented shoulder dysfunction after modified mastectomy with sectioning of the medial pectoralis nerve, while 6% presented shoulder dysfunction when the medial pectoralis nerve was preserved. Alpha and beta were estimated as 5% and 10%, respectively. Because of the lack of other studies, we used “shoulder dysfunction” as synonymous with loss of strength in the pectoralis major muscle and with loss of range of shoulder flexion and abduction for sample size estimation. For data analysis, Wilcoxon’s non-parametric test, Fisher’s exact test, Friedman’s analysis of variance and analysis of variance (ANOVA) were used.

## RESULTS

All of the women completed the physiotherapy sessions up to 43 days after surgery. The two groups of women were comparable with regard to age, body mass index (BMI; kg/m^2^), total lymph nodes, positive lymph nodes, duration of surgery and number of physiotherapy sessions (Table 1). 

More women in the group with preservation of the medial pectoral nerve suffered slight loss of muscle mass in the pectoralis major, as evaluated 15 and 43 days after mastectomy, compared with the group of women in whom this nerve was sectioned. However, these differences were not statistically significant (Table 2).

Preservation of the medial pectoral nerve was significantly associated with no loss of strength in the pectoralis major muscle 43 days after mastectomy, compared with the group of women in whom this nerve was sectioned. However, this difference had not been observed 15 days after surgery (Table 3). There were no significant differences with regard to loss of range of shoulder flexion and abduction between the 15^th^ and 43^rd^ days following surgery, between the groups of women in whom the medial pectoral nerve was preserved or sectioned (Tables 4 and 5).

**Table 1.** Distribution of the control variables studied, according to whether the medial pectoral nerve was preserved or sectioned

	n	Mean	SD	Range	P-value ^(a)^
Age (years)					
MPN preserved	16	50.3	11.9	31-74	0.59
MPN sectioned	14	53.1	14.2	35-76	
Body mass index (kg/m^2^)					
MPN preserved	16	27.4	5.5	20.6-40.1	0.66
MPN sectioned	12	27.3	3.7	22.8-36.1	
Total number of lymph nodes dissected					
MPN preserved	16	18.9	8.8	4.0-39.0	0.69
MPN sectioned	14	18.1	8.7	4.0-35	
Number of positive lymph nodes					
MPN preserved	11	6.2	9.0	1.0-30.0	0.61
MPN sectioned	9	7.7	10.9	1.0-35.0	
Duration of surgery (minutes)					
MPN preserved	11	101.4	51.0	50-195.0	0.70
MPN sectioned	10	83.5	25.5	60-150	
Number of physiotherapy sessions					
MPN preserved	16	20.0	1.3	17-21.0	0.96
MPN sectioned	14	20.0	1.5	17-21.0	

SD = standard deviation; MPN = medial pectoral nerve. (a) P-value, according to the Wilcoxon test.

**Table 2.** Physiotherapeutic evaluation of the proportional loss of muscle mass according to whether the medial pectoral nerve was preserved or sectioned (n = 30)

Time of evaluation (days after surgery)	Proportional loss of muscle mass (%)	Preserved		Sectioned		P ^(a)^
		n	(%)	n	(%)	
15	No loss	3	19	4	31	0.77
	Slight (1 to 25%)	7	44	3	23	
	Moderate (26% to 50%)	5	31	5	38	
	Considerable (51% or more)	1	6	1	8	
	Unknown	0		1		
43	No loss	2	13	2	15	0.42
	Slight (1 to 25%)	9	55	4	31	
	Moderate (26% to 50%)	3	19	6	46	
	Considerable (51% or more)	2	13	1	8	
	Unknown	0		1		

P-value, according to Fisher’s exact test.

**Table 3.** Physiotherapeutic evaluation of the proportional loss of muscle strength in the pectoralis major muscle at two different postsurgical times, according to whether the medial pectoral nerve was preserved or sectioned (n = 30)

Time of evaluation (days after surgery)	Proportional loss of strength (%)	Preserved		Sectioned		P ^(a)^
		n	(%)	n	(%)	
15	No loss	7	43	1	7	0.15
	Slight (1 to 25%)	1	6	2	14	
	Moderate (26% to 50%)	2	13	2	14	
	Considerable (51% or more)	6	38	9	64	
	Unknown	0		0		
43	No loss	13	81	4	31	0.03
	Slight (1 to 25%)	0	0	2	15	
	Moderate (26% to 50%)	1	6	3	23	
	Considerable (51% or more)	2	13	4	31	
	Unknown	0		1		

(a) P-value, according to Fisher’s exact test.

**Table 4.** Physiotherapeutic evaluation of the proportional loss of range of movement of the shoulder in abduction at two different postsurgical times, according to whether the medial pectoral nerve was preserved or sectioned (n = 30)

Time of evaluation (days after surgery)	Proportional loss of abduction (%)	Preserved		Sectioned		P ^(a)^
		n	(%)	n	(%)	
15	No loss	1	6	0	0	0.28
	Slight (1% to 25%)	2	13	5	36	
	Moderate (26% to 50%)	12	75	7	50	
	Considerable (51% or more)	1	6	2	14	
	Unknown	0		0		
43	No loss	1	6	0	0	0.43
	Slight (1% to 25%)	7	44	8	61	
	Moderate (26% to 50%)	8	50	4	31	
	Considerable (51% or more)	0	0	1	8	
	Unknown	0		1		

P-value, according to Fisher’s exact test.

**Table 5.** Evaluation of the proportional loss of range of flexion of the shoulder at two different postsurgical times, according to whether the medial pectoral nerve was preserved or sectioned (n = 30)

Time of evaluation (days after surgery)	Proportional loss of flexion (%)	Preserved		Sectioned		P ^(a)^
		n	(%)	n	(%)	
15	No loss	1	6	0	0	0.80
	Slight (1% to 25%)	9	56	9	64	
	Moderate (26% to 50%)	5	31	3	21	
	Considerable (51% or more)	1	6	2	15	
	Unknown	0		1		
43	No loss	0	0	1	8	0.67
	Slight (1% to 25%)	11	69	9	69	
	Moderate (26% to 50%)	5	31	3	23	
	Considerable (51% or more)	0	0	0	0	
	Unknown	0		1		

P-value, according to Fisher’s exact test.

## DISCUSSION

The patients who underwent modified radical mastectomy with preservation of the medial pectoral nerve presented significant reduction in the loss of strength in the ipsilateral pectoralis major compared with the patients who underwent mastectomy with nerve sectioning. However, no differences were found regarding atrophy of the pectoralis major muscle or loss of range of movement in the homolateral upper limb.

All the patients completed the physiotherapy program over the 43 days scheduled, making a total of 21 sessions. The results from the study were not affected by any occurrences of patients missing four or more sessions and there were no losses from the follow-up. One woman alone, who was in the group of patients whose medial pectoral nerve was sectioned, failed to appear for the final 43-day reevaluation visit, for personal reasons.

The range of movement in the upper limb and the strength of the pectoralis major muscle were measured in accordance with the definitions of good postural alignment and pure flexion and abduction, in which the patients were not allowed to compensate through other movements. This clearly hampered the procedure, particularly during the evaluation carried out on the 15^th^ day following surgery, when the recent scar tissue provoked significant pain and discomfort during the assessment.

Some difficulty was found in palpating the pectoralis major muscle, particularly during the evaluation carried out on the 15^th^ day after surgery, due to the recentness of the scarring, and in four cases on the 43^rd^ day after surgery, due to scar dehiscence. In two cases, a silicone prosthesis was used, which also technically hampered palpation of the pectoralis major muscle. Moreover, these forms of palpation did not allow evaluation of the thickness of the pectoralis major muscle. Such measurement would have provided a better indication of loss of muscle mass. In future studies, the use of other methods such as magnetic resonance imaging should be considered, in order to be able to objectively evaluate the changes in muscle mass in these patients.

Preservation of the medial pectoral nerve resulted in a smaller loss of muscle mass in the pectoralis major muscle, 15 and 43 days after mastectomy, compared with patients in whom this nerve was sectioned. Nevertheless, these differences were not significant. The sample size and the difficulty in objectively evaluating muscle mass may have been responsible for the failure of this study to observe any difference. The way in which the muscle mass in the pectoralis major muscle should be evaluated in women in whom the medial pectoral nerve has been damaged has not yet been well established. A previous study that evaluated this relationship failed to describe how atrophy of the pectoral muscle was evaluated, although it was accepted that this occurs frequently when the pectoral nerve is sectioned during mastectomy.^14^

In our study, 4 out of the 20 women who had been randomized to the preservation group were excluded because of accidental injury of the medial pectoral nerve and the presence of lymph nodes adhering to it. It is important to emphasize that preservation of the medial pectoral nerve is not a simple procedure to perform. Although recommended by Patey,^11^ cases of accidental injury of this nerve must be taken into consideration. We were unable to find any data in the literature on the frequency of such injuries, in order to make comparisons with the findings of the present study. The same type of lesion, whether accidental or not, may also occur in Madden’s mastectomy, in which both pectoral muscles are preserved, and in quadrantectomy, during level II axillary dissection. Merson et al.^14^ indirectly evaluated the sectioning of the medial pectoral nerve by sectioning the pectoralis minor muscle, and observing the associated complications. They obtained significant data regarding the differences in muscle mass between the two types of surgical technique studied.

There was no difference in the number of lymph nodes dissected between the group in which the medial pectoral nerve was preserved and the group in which it was sectioned. Similar results were reported from the study carried out by Merson et al.,^14^ and these findings confirm the notion that preservation of the medial pectoral nerve is safe in oncological terms.

## CONCLUSION

Preservation of the medial pectoral nerve, compared with sectioning the nerve, among women undergoing modified mastectomy, was feasible. Forty-three days after surgery, this technique resulted in smaller loss of muscle strength in the pectoralis major muscle, had no effect on muscle mass in the pectoralis major muscle, and did not cause any change in the range of movement of abduction or flexion of the homolateral upper limb.
